# Bladder lymphoepithelioma-like carcinoma: a case report

**DOI:** 10.1093/jscr/rjac209

**Published:** 2022-05-06

**Authors:** Moez Rahoui, Yassine Ouanes, Kays Chaker, Kheireddine Mrad Dali, Mokhtar Bibi, Ahmed Sellami, Sami Ben Rhouma, Yassine Nouira

**Affiliations:** Urology Department La Rabta Hospital, Tunis, Tunisia; Urology Department La Rabta Hospital, Tunis, Tunisia; Urology Department La Rabta Hospital, Tunis, Tunisia; Urology Department La Rabta Hospital, Tunis, Tunisia; Urology Department La Rabta Hospital, Tunis, Tunisia; Urology Department La Rabta Hospital, Tunis, Tunisia; Urology Department La Rabta Hospital, Tunis, Tunisia; Urology Department La Rabta Hospital, Tunis, Tunisia

## Abstract

Bladder lymphoepithelioma-like carcinoma is a rare entity. It represents a particular variant of urothelial carcinoma characterized by an important infiltrating power. Therapeutic management of this cancer is not codified. Surgery associated with chemotherapy seems to be the best therapeutic option. Few cases of this tumor have been reported in the literature. We report a case of bladder lymphoepithelioma-like carcinoma in a 52-year-old patient who presented with gross hematuria and discusses difficulties of diagnostic and treatment.

## INTRODUCTION

Bladder cancer is the ninth most frequently diagnosed cancer in the world and the second most common urological cancer after prostate cancer, with a clear male predominance [1]. The most common histological type is urothelial carcinoma. It represents 90% of bladder tumors. However, several specific histological variants have been reported in the literature [2]. Lymphoepithelioma-like carcinoma (LELC) is a rare variant of infiltrating urothelial carcinoma first described in the bladder by Zukerberg et al. in 1991 [2]. We herein report a new case of bladder LELC in a 52-year-old patient who presented with gross hematuria and discusses difficulties of diagnostic and treatment.

## CASE REPORT

**Figure 1:**
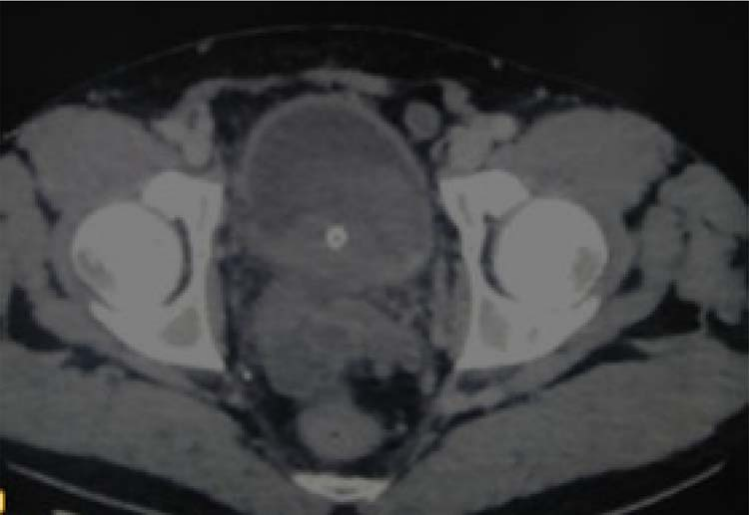
CT revealing a bladder tumor of 6 × 5 cm over the trigone and left wall.

**Figure 2:**
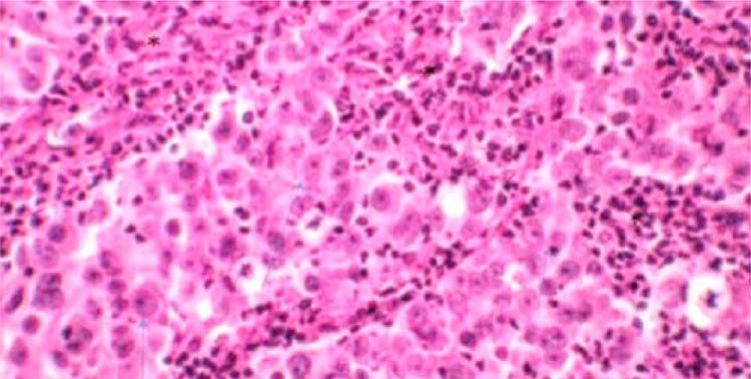
Microscopic aspect of bladder LELC.

**Figure 3:**
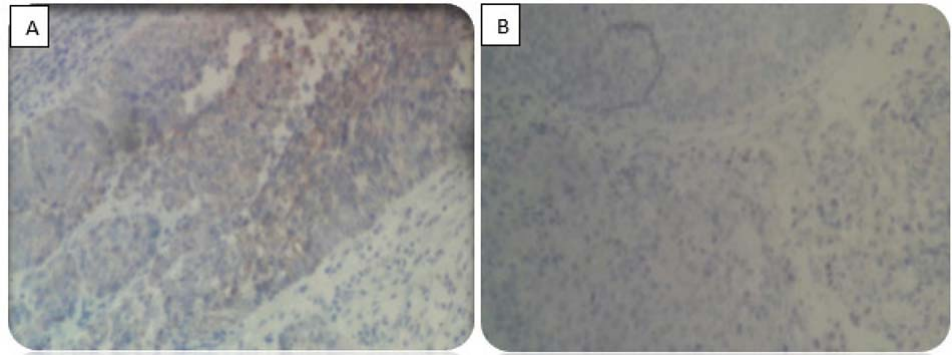
Immunohistochemical study. (**A**) Positive staining for CKAE1/AE3. (**B**) Negative staining for CD7 and CD20.

A 52-year-old man, diabetic, presented for gross hematuria associated with pelvic pain. His physical examination was normal. Ultrasonography and chest and abdominopelvic computed tomography (CT) scan revealed a urinary bladder tumor of 6×5 cm over the trigone and the left wall with left hydronephrosis (Fig. 1). Cystoscopic exploration revealed a solid lesion in the left wall and the trigone with a large base, infiltrating the left meatus. The patient underwent a complete transurethral resection of the bladder tumor. Histological examination showed a tumor proliferation made of nests and patches of large cells with eosinophilic cytoplasm and atypical and nucleated nuclei, infiltrating the bladder muscle. Tumor stroma is loaded with lymphoid elements (Fig. 2). The immunohistochemical study showed that this proliferation was negatively stained for (CK7 and CK20) but positively stained by epithelial markers (AE1/AE3). The final histopathological diagnosis was LELC (Fig. 3). A chest and abdominopelvic CT scan did not show pelvic lymphadenopathy or secondary location. Radical surgery was then decided without neoadjuvant therapy. Cystoprostatectomy with Bricker diversion and lymph node dissection was performed. The postoperative course was uneventful. The pathological examination of the surgical specimen confirms the diagnosis of a bladder LELC invading the perivesical fat. The patient was referred to the medical oncology department, where he received cisplatin-based chemotherapy. After 8 months of clinical and radiological check-ups, there was no functional complaint or any sign of reoccurrence.

## DISCUSSION

Lymphoepitheliomas represent undifferentiated malignant epithelial tumors of the nasopharynx characterized by a prominent lymphoid infiltrate [1]. LELCs have similar histological features but develop outside the nasopharynx [2]. Urinary location is exceptional, with the bladder being the most frequent site. LELC of the bladder was first described in 1991 by Zukerberg et al. [2] and represents 0.4–1.3% of all bladder carcinomas with a male predominance (70.9%) [2]. In the literature, >150 cases of LELC of the bladder have been described [2]. Because of the rarity of this tumor, its management is not codified.

Macroscopic hematuria is the most common symptom and has been reported in the majority of cases. The stage at diagnosis of those tumors was in 87% of cases T2–T3 stage [3].

To date, the exact pathogenesis of this tumor is not well established. Although the association between lymphoepithelioma of the nasopharynx and other tissues (lung, stomach, thymus and salivary gland) and Epstein–Barr virus has already been proven, in the bladder, hybridization with Epstein–Barr virus-encoded RNA has been reported to be consistently negative in different series [3].

Some authors have suggested that abnormalities in p53 regulation have a role in the pathogenesis of these tumors [2, 3]. Other authors have suggested a link between Bacillus Calmette-Guerin (BCG) therapy and LELC of the urinary bladder [3].

Histologically, bladder LELCs are classified according to the amount of lymphoepithelial tissue within the tumor, into pure (100%), predominant (50–99%) and focal (<50%), admixed with typical urothelial carcinoma, squamous cell carcinoma or adenocarcinoma [2]. Immunohistochemistry is essential for differential diagnosis and usually reveals positivity of tumor cells for various broad spectrum epithelial markers, such as GATA3, epithelial membrane antigen, AE1/AE3, CK7 and CK8; in contrast, CK20 staining is frequently negative [2].

Given the rarity of this tumor, there is no codified treatment allowing a better survival. Several treatments have been proposed but it turns out that a multimodal treatment seems necessary. Some authors have proposed transurethral resection [4]. However, others have shown that radical cystectomy represents the reference treatment [5]. Adjuvant radiochemotherapy has been proposed after conservative management or radical surgery. However, most studies advocate routine use of cisplatin-based chemotherapy as adjuvant therapy after transurethral resection or cystectomy [2, 4, 5].

## CONCLUSION

Bladder LELC is a rare clinical entity with undefined therapeutic strategies. Radical cystectomy seems the best therapeutic option, especially in patients with cancer infiltrating the muscle. However, more studies and experiences are needed to establish clear guidelines of this rare entity.

## CONFLICT OF INTEREST STATEMENT

The authors declare that there are no conflicts of interest regarding the publication of this article.
